# A Kinetic Map of the Influence of Biomimetic Lipid Model Membranes on Aβ_42_ Aggregation

**DOI:** 10.1021/acschemneuro.2c00765

**Published:** 2022-12-27

**Authors:** Kevin N. Baumann, Greta Šneiderienė, Michele Sanguanini, Matthias Schneider, Oded Rimon, Alicia González Díaz, Heather Greer, Dev Thacker, Sara Linse, Tuomas P. J. Knowles, Michele Vendruscolo

**Affiliations:** † Yusuf Hamied Department of Chemistry, University of Cambridge, CambridgeCB2 1EW, U.K.; ‡ Cavendish Laboratory, University of Cambridge, CambridgeCB3 0HE, U.K.; § Department of Biochemistry and Structural Biology, Lund University, LundSE22100, Sweden

**Keywords:** Alzheimer’s disease, amyloid β, protein aggregation, lipid membranes, aggregation kinetics, cryo-electron microscopy

## Abstract

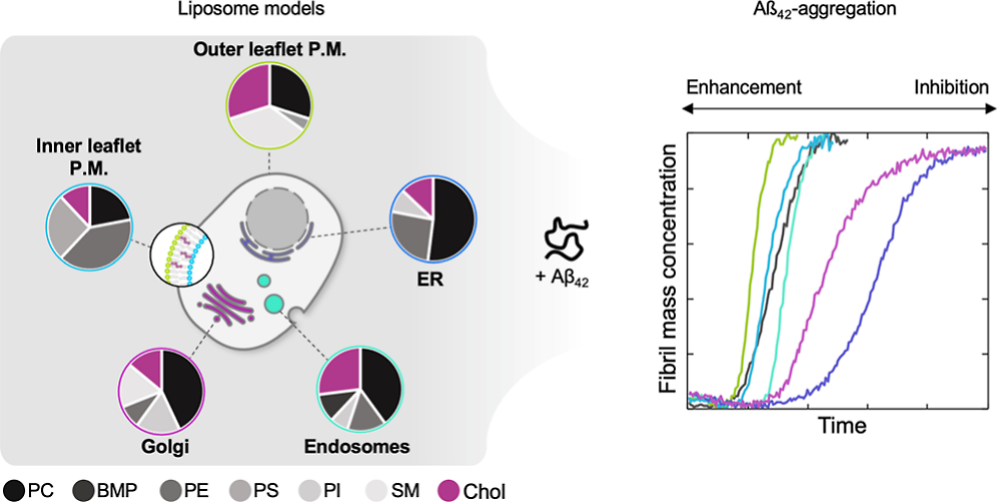

The aggregation of the amyloid β (Aβ) peptide is one of the molecular hallmarks of Alzheimer’s disease (AD). Although Aβ deposits have mostly been observed extracellularly, various studies have also reported the presence of intracellular Aβ assemblies. Because these intracellular Aβ aggregates might play a role in the onset and progression of AD, it is important to investigate their possible origins at different locations of the cell along the secretory pathway of the amyloid precursor protein, from which Aβ is derived by proteolytic cleavage. Senile plaques found in AD are largely composed of the 42-residue form of Aβ (Aβ_42_). Intracellularly, Aβ_42_ is produced in the endoplasmatic reticulum (ER) and Golgi apparatus. Since lipid bilayers have been shown to promote the aggregation of Aβ, in this study, we measure the effects of the lipid membrane composition on the in vitro aggregation kinetics of Aβ_42_. By using large unilamellar vesicles to model cellular membranes at different locations, including the inner and outer leaflets of the plasma membrane, late endosomes, the ER, and the Golgi apparatus, we show that Aβ_42_ aggregation is inhibited by the ER and Golgi model membranes. These results provide a preliminary map of the possible effects of the membrane composition in different cellular locations on Aβ aggregation and suggest the presence of an evolutionary optimization of the lipid composition to prevent the intracellular aggregation of Aβ.

## Introduction

The cytotoxic aggregation of amyloid β (Aβ) is considered a major contributor to the onset and progression of Alzheimer’s disease (AD).^1−3^ While neurotoxic fibrillar deposits are found extracellularly, oligomeric species of Aβ are found intracellularly and in various cellular organelles, probably originating from a combination of local proteolytic processing, escape from the secretion pathway, and re-uptake of extracellular peptides.^4−7^ As Aβ is a product of the proteolysis of the membrane-bound amyloid precursor protein (APP), the influence of lipid membrane compositions on the aggregation of Aβ is of particular interest. Indeed, different membrane compositions have been shown to affect the proteolytic processing of APP and the kinetics of Aβ aggregation.^8−16^ Furthermore, cholesterol has been shown to increase the primary nucleation rate of Aβ in model cell membranes,^15^ an effect that is dependent on the composition of the lipid membranes themselves. It is also known that, while individual lipids can either accelerate or delay the aggregation of Aβ according to their physicochemical properties, mixtures of lipids can average out these effects and protect against aggregation.^16^ This phenomenon, which can be referred to as “resilience in complexity”,^16^ is of particular interest given that biological membranes exhibit a great repertoire of lipids and the membranes of organelles are constituted by individual and optimized lipid compositions.^17,18^ Therefore, because of the broad distribution of the individual effects lipid species composing a lipid membrane have on the aggregation of Aβ and other amyloid species,^16,19^ the net effect on Aβ aggregation may be small. Yet, different compositions might result globally in either an enhancement or inhibition of the aggregation speed.

**Figure 1:**
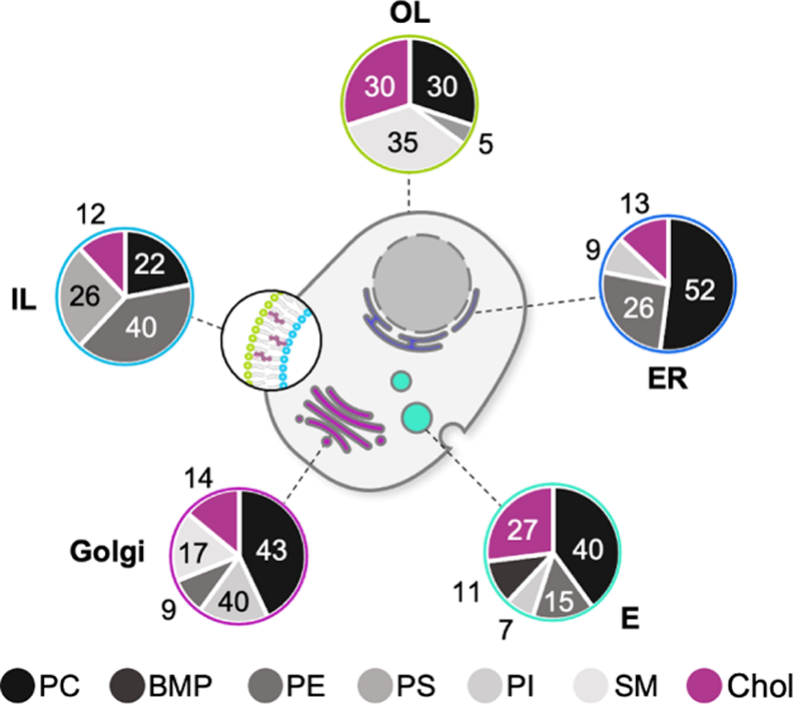
Lipid compositions of the chosen model membranes. LUVs with the model lipid membrane compositions OL: outer leaflet of the plasma membrane, IL: inner leaflet of the plasma membrane, E: late endosomes, G: Golgi apparatus, ER: endoplasmic reticulum were formed. The contents of the individual lipid types are indicated in %.

In this study, we investigate the effects of lipid membrane compositions on Aβ_42_ aggregation using large unilamellar vesicles (LUVs) that mimic the composition of cellular components that have been implicated in Aβ processing and secretion: the outer and inner layers of the plasma membrane, late endosomes, the Golgi apparatus, and the ER. We selected the 42 amino acid residue peptide as it is the dominant species found in senile plaques and is therefore suggested to be linked to the onset and progression of AD.^3,20,21^ Further studies also indicate that Aβ_42_ has a higher propensity for aggregation in comparison to shorter Aβ alloforms and that oligomeric products can be found intracellularly.^5,7^ To obtain a physiologically relevant model for each lipid membrane, we selected the most prevalent lipid types as found in the literature^17,22^ (mol % > 5%, Table 1, Figure 1) and used complex mixtures of fatty acidic chains from commercially available purified brain lipid extracts. We analyzed the effects of the interaction of Aβ with the LUVs and the resulting aggregation by a combination of fluorescence assays and cryo-electron microscopy (cryo-EM).

**Table 1:** Lipid Mixtures Mimicking the Lipid Membrane Compositions of Different Cellular Compartments^a^

	lipid composition (mol %)						
cellular compartment	PC	PE	PI	PS	SM	BMP	Chol
outer leaflet of the plasma membrane*	30			5	35		30
inner leaflet of the plasma membrane*	22	40		26			12
late endosomes**	40	15			7	11	27
Golgi**	43	17	9		17		14
ER**	52	26	9				13

a Lipid compositions according to *: Lorent et al.,^22^ **: van Meer et al.^17^ Lipid types: PC (phosphatidylcholine), PE (phosphatidylethanolamine), PI (phosphatidylinositol), PS (phosphatidylserine), SM (sphingomyelin), BMP (bis(monoacyl-glycero)phosphate), chol (cholesterol).

## Results and Discussion

**Figure 2:**
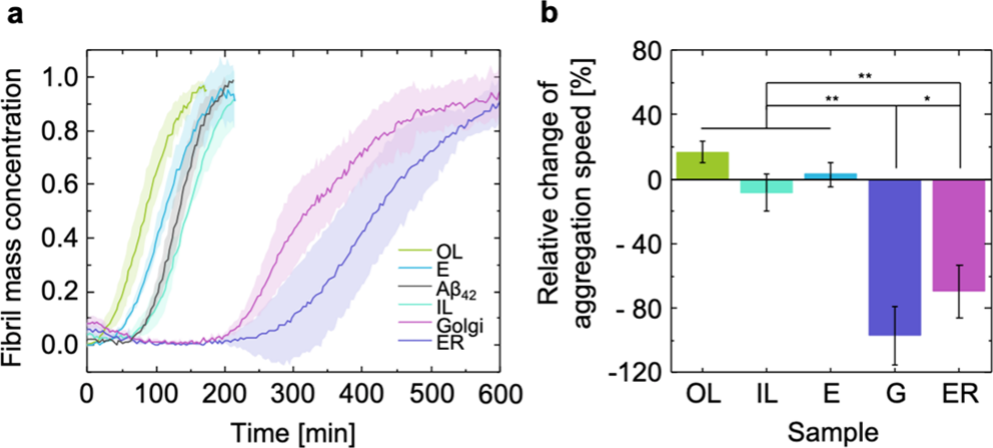
Aggregation kinetics of Aβ_42_ in the presence of different lipid model membranes. (a) Set of averaged traces of the Aβ_42_ aggregation kinetics in the presence of lipid model membranes monitored using a ThT fluorescence assay (*n* ≥ 3). The half time (*t*_1/2_) was calculated for each membrane composition (100 μM lipid concentration) and compared to that of Aβ_42_ in solution (2 μM). (b) Using *t*_1/2_ as the reference, the enhancement or inhibition of the aggregation kinetics imposed by the individual membrane compositions was measured. Two-way ANOVA reveals a statistically significant difference between the *t*_1/2_ of G and ER (*p* = 0.041), both of which are statistically significantly different from OL (G: *p* = 0.001, ER: *p* = 0.001), IL (G: *p* = 0.001, ER: *p* = 0.009), and E (G: *p* = 0.001, ER: *p* = 0.001). Error bars show the pooled standard deviation of at least three independent data sets per lipid membrane model. OL: outer leaflet of the plasma membrane, IL: inner leaflet of the plasma membrane, E: late endosomes, G: Golgi apparatus, ER: endoplasmic reticulum.

The aggregation kinetics of Aβ_42_ in the presence of different membrane-mimetic LUVs was monitored using the amyloid-sensitive dye thioflavin T (ThT) in a fluorescence assay (Methods). Our results show that lipid membranes, with compositions corresponding to the organelles involved early in the secretory pathway of APP, inhibit the aggregation of the Aβ_42_ peptide, with aggregation half-time values for ER and Golgi that are up to twice the ones of Aβ_42_ alone (Figure 2). Conversely, the lipid model membranes corresponding to the outer leaflet of the plasma membrane accelerated the aggregation of the Aβ_42_ peptide by approximately 20% in comparison to Aβ_42_ alone. The lipid model membranes of the inner leaflet of the plasma membrane, on the other hand, exhibit a slightly inhibiting effect, and the model membranes corresponding to late endosomes cause no effect or even a minor enhancement in amyloid formation. In the early stage of endosomal maturation, the lipid membrane composition of the inner leaflet of endosomal membranes is comparable to that of the outer leaflet of the plasma membrane. With ongoing maturation, the membrane composition of endosomes alienates further from the initial state, while concurrently the pH decreases.^17^ In our studies, we did not observe any pH dependence of the effects of the endosomal lipid model membranes on the aggregation of Aβ_42_ (Supporting Information, Figure S2).

**Figure 3:**
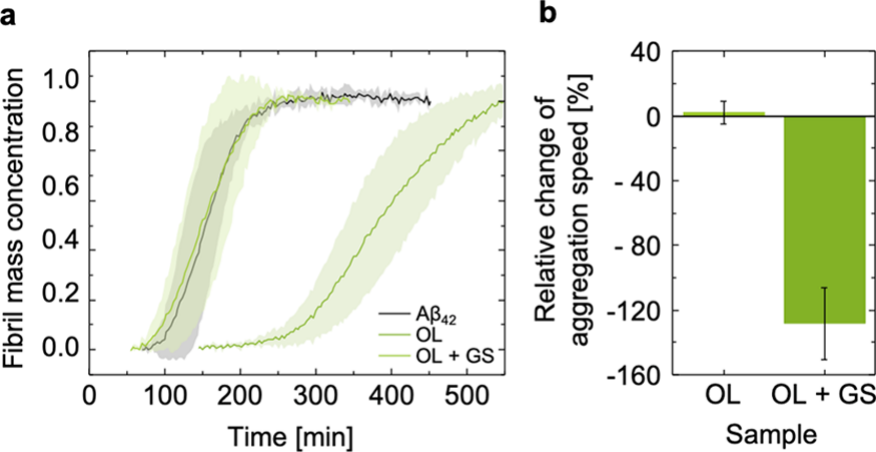
Aggregation kinetics of Aβ_42_ in the presence of the OL model membranes with and without gangliosides. (a) Set of averaged traces of the Aβ_42_ aggregation kinetics in the presence of the model lipid membranes monitored through a ThT fluorescence assay (*n* = 5). (b) *t*_1/2_ variation was calculated for the membrane compositions OL and OL + GS (100 μM lipid concentration) in relation to Aβ_42_ in solution (2 μM). Using *t*_1/2_ of Aβ_42_ alone as the reference, the OL + GS model membranes appear to inhibit the Aβ_42_ aggregation. Error bars show the standard deviation of five traces.

Of particular interest was the influence of gangliosides in the OL membranes. To address this question, gangliosides^23^ (from porcine brain extract) were added at a 20% molar ratio to the OL model membranes, yielding OL + GS. While lipid membranes can be saturated with gangliosides at around a 10% molar ratio,^24^ these samples potentially contain a mixture of ganglioside-saturated membranes and ganglioside micelles. The presence of the gangliosides increased the aggregation half time substantially by more than twice the *t*_1/2_ of the Aβ_42_ peptide (Figure 3).

**Figure 4:**
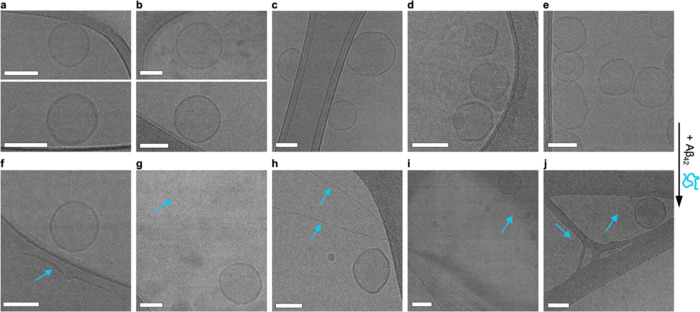
Cryo-EM reveals no morphological influence of Aβ_42_ aggregation on the LUVs. (a–e) Model lipid membranes of: Golgi (a), ER (b), endosomes (c), and the inner (d) and outer leaflets (e) of the plasma membrane before the incubation with Aβ_42_ overnight. Endosomes, inner leaflet model, and outer leaflet model lipid membranes show organization of the lipid membrane into facets. (f–j) After the addition of Aβ_42_, amyloid fibrils can be seen in all samples (blue arrows). Scale bars: 100 nm.

We next investigated whether Aβ_42_ aggregation modifies the morphology of the model lipid membranes. Using cryo-EM, model lipid membranes of the outer leaflet, inner leaflet, and endosomes presented a distinctly facetted surface, most likely due to the organization of the lipids into phase domains, where specific lipid species dominate the local membrane composition (Figure 4a–e). Longer fibrils were found in the samples containing the model lipid membranes of the outer leaflet and late endosomes, a possible effect of their effective enhancement of the aggregation kinetics. We found no morphological influence of the aggregation of the peptide on the LUVs, regardless of their lipid composition (Figure 4f–j). Furthermore, no or only weak association was found between the Aβ_42_ fibrils and the LUVs (Figure 4f–j). This agrees with previous findings where TEM images of fibrils in the presence of LUVs with complex lipid mixtures showed no morphological changes and only weak association between fibrils and LUVs.^10,16^

**Figure 5:**
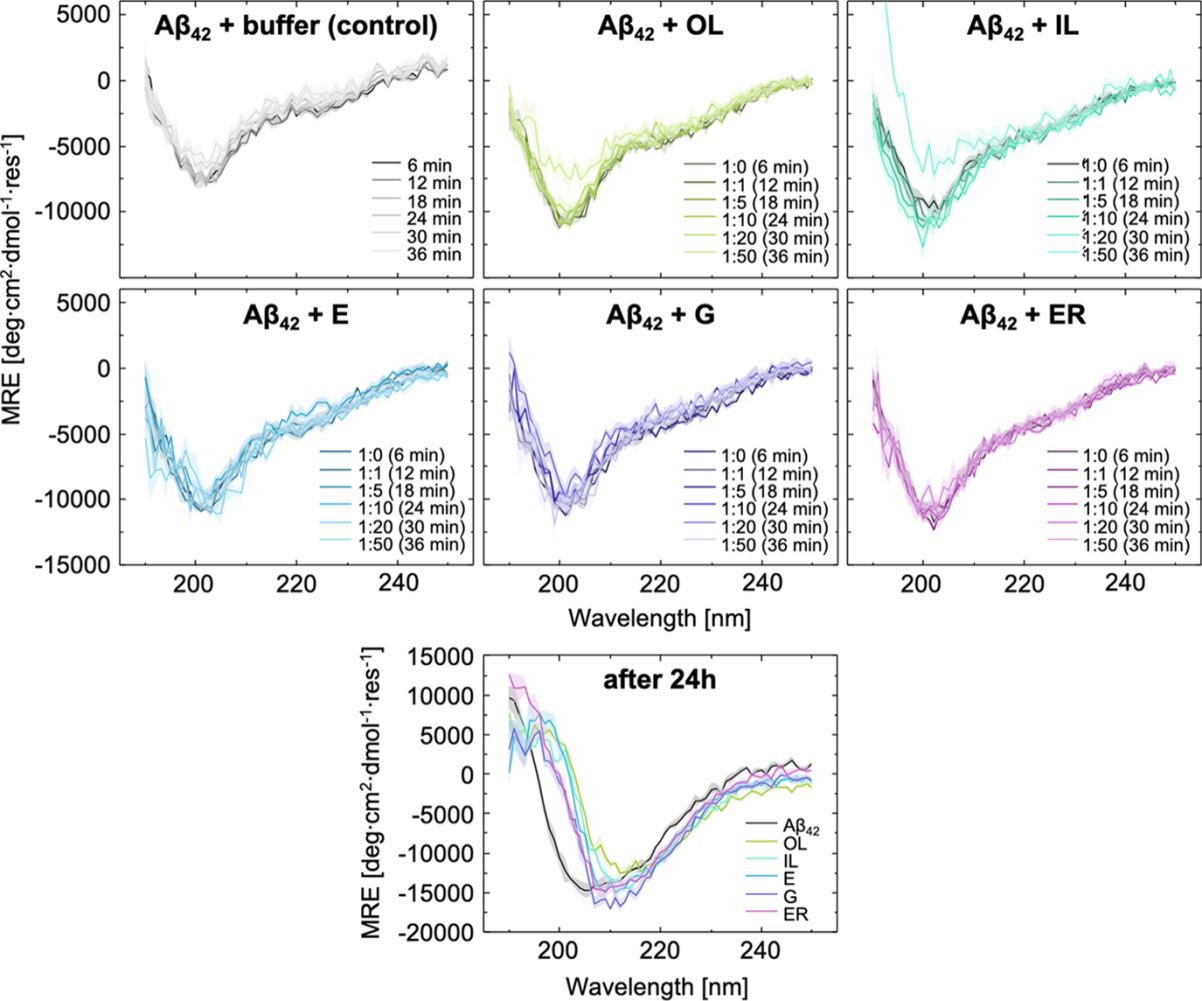
Aβ_42_ does not change its secondary structure in the presence of varying concentrations of LUVs. CD data of Aβ_42_ incubated with the model lipid membranes at increasing concentrations. After 24 h (Aβ_42_ and LUVs ratio 1:50), the shift of the minima by approximately 10 nm indicates β-sheet formation, enhanced by the model lipid membranes.

**Figure 6:**
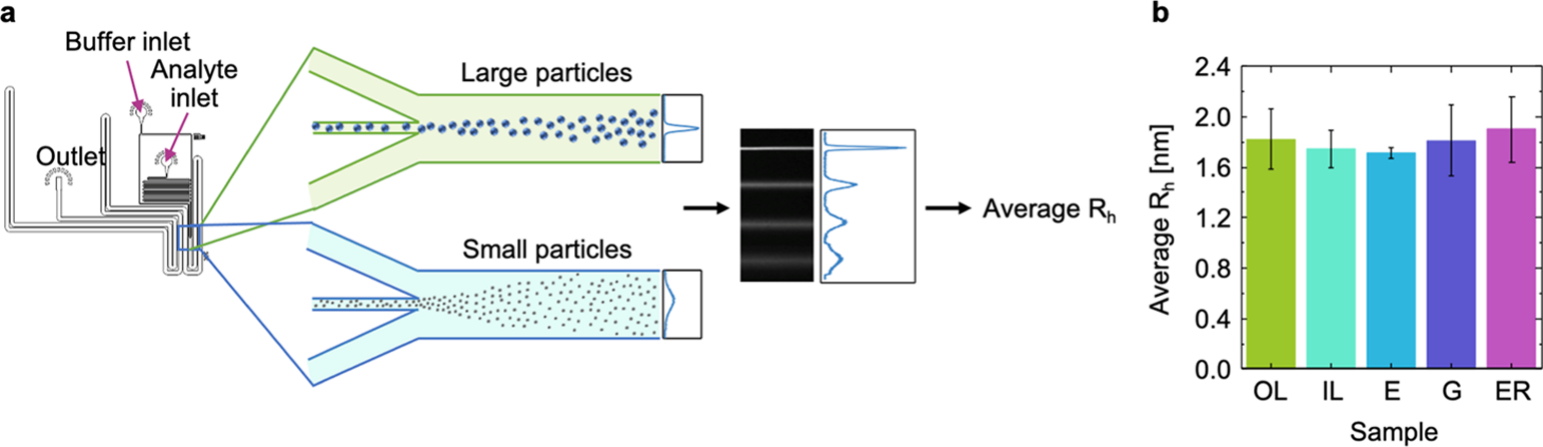
Microfluidic diffusional sizing measurements of the affinity of Aβ_42_ monomers to the lipid model membranes. Upon binding to a LUV, the average hydrodynamic radius (*R*_h_) of fluorescently-labeled Aβ_42_ is expected to increase by the radius of the LUVs. However, no increase in the *R*_h_ of fluorescently labeled monomeric Aβ_42_ upon mixing with the LUVs could be detected, indicating that monomeric Aβ_42_ does not detectably interact with model lipid membranes. (a) Principle of the microfluidic diffusional sizing method: the higher diffusion rates of small particles lead to a broader distribution across the channel width at the detection region. (b) Average *R*_h_ of fluorescently labeled Aβ_42_ monomers in a mixture with the model lipid membranes was back-calculated based on these diffusion profiles. Error bars indicate the standard deviation (*n* = 3).

The lack of strong interactions between LUVs and fibrils suggests that the LUVs facilitate the aggregation via a catalytic mechanism that involves interaction with smaller species rather than fibrils. According to this mechanism, the LUV membranes might promote the aggregation of Aβ_42_ by transiently binding small species, thereby facilitating nucleation through locally increased concentrations.^25−27^ Another possibility would be that oligomers bound to lipid membranes would dissociate less readily, thus having more time to undergo the conformational conversion step observed in the aggregation of Aβ_42_.^28^ In order to investigate this possibility, we asked whether lipid membranes induce a stable secondary structure of Aβ_42_, as one would expect in a scenario where lipid surfaces would induce peptide ordering.^29^ We monitored the evolution of circular dichroism (CD) spectra of Aβ_42_ in the presence of LUVs. Background-subtracted CD data show that Aβ_42_ does not change its secondary structure in the presence of varying concentrations of LUVs in the first hour (Figure 5). Within this time, the peptide is expected to be present in mainly a monomeric state, according to the kinetic profiles obtained from the ThT assays. To further corroborate this possibility, we used microfluidic diffusional sizing to probe the binding of monomeric Aβ_42_ to the LUVs. Similarly to CD, no binding events were detected using this method (Figure 6). The experiments could not detect small oligomers bound to the lipid membranes, as suggested by previous studies,^25,30^ and determine whether these bound oligomers are missing from an available pool of aggregation seeds or if they further enhanced the aggregation kinetics by initiating secondary pathways.^21^

In conclusion, our results imply that the aggregation of Aβ_42_ is inhibited by model membranes mimicking the lipid composition of Golgi and ER membranes. The only model membrane type that generated a tendency toward the enhancement of the aggregation kinetics was that of the outer leaflet of the plasma membrane. These observations support the theory of a possible evolutionary pressure toward the optimization of the membrane compositions of organelles in the early stages of the APP secretory pathway to avoid the intracellular aggregation of Aβ_42_.^7,31^

## Experimental Section

### LUV Preparation

LUVs were prepared as previously described^16^ by extruding lipid solutions at 500 μM concentration suspended in a solution containing 20 mM NaHPO_4_ buffered with 0.2 mM EDTA (pH = 8.0) through 100 nm extrusion membranes (Avanti Lipids) after sonication at 40 °C for 30 min. All lipids, including those obtained from brain extracts, were purchased from Avanti and stored in chloroform. The LUV compositions for the model membranes are gathered in Table 1.

### Aβ_42_ Purification

The recombinant Aβ(M1-42) peptide (M DAEFRHDSGY EVHHQKLVFF AEDVGSNKGAIIGLMVGGVVIA), here referred to as Aβ_42_, was expressed in the *Escherichia coli* BL21-Gold(DE3) strain (Stratagene, USA) and purified as described previously with slight modifications.^16,32^ Briefly, the transformed *E. coli* cells were sonicated, and the extracted inclusion bodies were dissolved in 8 M urea. The solution was then ion exchanged in batch mode on diethylaminoethyl cellulose resin and lyophilized. These lyophilized fractions were further purified using a Superdex 75 HR 26/60 column (GE Healthcare, USA), and the eluates were analyzed using SDS-PAGE to confirm the presence of the desired protein product. The fractions containing the recombinant protein were pooled, aliquoted, frozen using liquid nitrogen, and lyophilized again to obtain the working stock.

### ThT Assay

In order to prepare a solution of pure monomeric peptide, the lyophilized Aβ_42_ peptide was resuspended in 6 M guanidinium hydrochloride (GuHCl) and then purified from excess salt and potential oligomeric species using gel filtration on a size exclusion column (Superdex 75 10/300 GL, GE Healthcare) at a flow rate of 0.5 mL/min and eluted in 20 mM sodium phosphate and 0.2 mM EDTA buffer (at pH 8.0). The center of the peak was collected, and the peptide concentration was determined from the averaged concentration using the Lambert–Beer equation
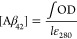
where OD is the optical density at 280 nm measured at the start and at the peak of the collection, ε_280_ is the molar absorptivity coefficient at 280 nm (for Aβ_42_, ε_280_ = 1490 M·cm^–1^), and *l* = 2 mm is the optical path length. The obtained peptide was diluted to the desired concentration of 2 μM with 20 mM sodium phosphate and 0.2 mM EDTA buffer (pH 8.0) and supplemented with 20 μM ThT and 100 μM LUVs. All samples were prepared in low-binding test tubes (Eppendorf, Hamburg, Germany) on ice. Each sample was then pipetted into multiple wells of a 96-well half-area, low-binding, clear bottom, and PEG coated plate (Corning 3881, Corning, New York, NY, USA). Assays were initiated by placing the 96-well plate at 37 °C under quiescent conditions in a plate reader (Fluostar Omega or Fluostar Optima, BMG Labtech). The ThT fluorescence was measured through the bottom of the plate with a 440 nm excitation filter and a 480 nm emission filter. The aggregation half time was extracted from the fibril mass concentration, which was calculated using the formula below.
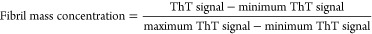


### Cryo-Electron Microscopy

Specimens were prepared by plunge freezing suspensions (at the original concentration) on copper grids (300 mesh) containing lacey carbon film. Prior to use, the grids were glow discharged using a Quorum Technologies GloQube instrument at a current of 25 mA for 60 sec. The sample (3 μL) was pipetted onto a TEM grid, blotted for 3 sec at blot force -5 using dedicated filter paper, and immediately plunged into liquid ethane using a Vitrobot Mark IV. The Vitrobot chamber was set to 4 °C and 95% humidity. Specimens after vitrification were kept under liquid nitrogen until they were inserted into a Gatan Elsa cryo holder and imaged in the TEM at −178 °C. Images were collected using a Thermo Scientific (FEI) Talos F200X G2 microscope at 200 kV at low dose using a Ceta 16M CMOS camera.

### Circular Dichroism

Far-UV CD spectra were recorded between 190 and 250 nm using a Chirascan system (Applied Photophysics). A solution of 15 μM Aβ_42_ was transferred to a quartz cell with a 0.1 cm path length and incubated at 25 °C. After six minutes, a spectrum was recorded as ellipticity θ (in mdeg). Following the initial measurement, a portion of buffer (control) or LUV solution was added to achieve a 1:1 Aβ_42_/LUV molar ratio, and 6 min after the first measurement, the spectrum was recorded again. Every 6 minutes thereafter, a spectrum with increasing amounts of LUVs was recorded. Three spectra per time point were averaged, corrected by subtracting the buffer spectrum, and normalized to mean residue ellipticity (MRE; in deg·cm^2^·dmol^–1^) using the sample concentration (in M) at that time point and the number of residues in the protein
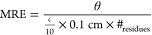


Finally, the spectra of all samples at 24 h following the initial measurements (all at the final molar ratio of 1:50 Aβ_42_/LUVs) were recorded to reflect fully aggregated controls.

### Aβ_42_ Y10C Purification and Labeling for Microfluidic Diffusional Sizing

The Aβ_42_ Y10C mutant was purified as described above, except that 1 mM DTT was added to all buffers. Lyophilized fractions (∼14 μM) of the peptide were dissolved in 50 μL of deionized water. Alexa fluor 488 was added to the dissolved peptide in excess and kept overnight at 4 °C for labeling. The following morning, the mix was added in 1 mL of 6 M GuHCl, 20 mM sodium phosphate, 0.2 mM EDTA, and pH 8.5 solution and subjected to gel filtration on a Superdex 75 10/300 column in 20 mM sodium phosphate buffer pH 8.0 with 0.2 mM EDTA. Absorption at wavelengths of 280 and 488 nm was monitored to follow the elution of the labeled peptide and to monitor any unlabeled peptide, if present. The aliquots collected from the SEC were then stored at −80 °C until further use.

### Microfluidic Diffusional Sizing

A master mold for the production of the polydimethylsiloxane (PDMS)-based microfluidic diffusional sizing devices was generated using UV soft-lithography. Briefly, a negative photoresist (SU8-3050) was spin-coated on a silicon wafer to yield a 25–50 μm layer. The silicon wafer was then baked for 10 min at 95 °C on a hot plate. The wafer was then exposed to UV light through a photomask, defining the channel geometries for 40 s. After exposure, the wafer was baked for 5 min at 95 °C, followed by development in a propylene glycol methyl ether acetate bath. The correct height of the features was measured with a profilometer.

To fabricate microfluidic devices, PDMS was mixed with carbon nanopowder (Sigma, USA) and a curing agent at a 10:1 mass ratio. The mixture was then centrifuged for 45 min at 5000 rpm, poured on the master, and degassed under vacuum. Subsequently, the devices were baked for 1 h at 65 °C. Cured PDMS chips were then peeled off the master. A biopsy puncher was used to make channel inlets and outlets, followed by device bonding to the glass slides. To this end, oxygen plasma treatment was used to activate the PDMS and glass surfaces.

Microfluidic diffusional sizing experiments were carried out as previously described.^33^ Before the measurements, the surface of microfluidic diffusional sizing devices was pre-treated with 0.01% Tween 20. 2.5 μM of the Alexa 488-labeled Aβ_42_ Y10C mutant was mixed with 100 μM LUVs in a 20 mM NaHPO_4_, 0.2 mM EDTA (pH = 8.0) buffer and flown in a 25 μM diffusional sizing device at a 50 μL/h flow rate. The devices were equilibrated for 5 min before recording fluorescent traces across channels. To obtain hydrodynamic radii, the images were analyzed with a custom-written Python script, utilizing the rate laws of diffusive mass transport under laminar flow conditions.

### Dynamic Light Scattering

The LUVs were assessed regarding their monodispersity and average hydrodynamic diameter by dynamic light scattering (DLS) after production. The vesicles were measured at their native concentration using a Zetasizer Nano (Malvern, UK) and Zen0040 disposable cuvettes. The measurements were performed at room temperature. The values can be reviewed in the Supporting Information, Figure S1.

## Supporting Information Available

Hydrodynamic diameters of the liposomal model membranes measured by DLS, and the effect of pH on the aggregation of Aβ_42_ in the presence of late endosomal model membranes (PDF)
