# Supplementary material for: A Kinetic Map of the Influence of Biomimetic Lipid Model Membranes on Aβ42 Aggregation
Source: ACS Chem Neurosci. 2022 Dec 27;14(2):323–9. doi: 10.1021/acschemneuro.2c00765 (PMC9853501; doi:10.1021/acschemneuro.2c00765)
Supplement: Supplementary file 1 — cn2c00765_si_001.pdf [file cn2c00765_si_001.pdf]

Supporting Information - A Kinetic Map of the Influence of Model Intracellular Lipid Membranes on Aβ_{42} Aggregation

Kevin N. Baumann[1], Greta Šneiderienė[1], Michele Sanguanini[1], Matthias Schneider[1], Oded Rimon[1], Alicia González Díaz[1], Heather Greer[1], Dev Thacker[3], Sara Linse[3], Tuomas P. J. Knowles[1,2], Michele Vendruscolo[1]*

[1]University of Cambridge, Yusuf Hamied Department of Chemistry, Cambridge CB2 1EW, United Kingdom

[2]University of Cambridge, Cavendish Laboratory, Cambridge CB3 0HE, United Kingdom

[3]Lund University, Department of Biochemistry and Structural Biology, SE22100 Lund, Sweden

*Correspondence to: mv245@cam.ac.uk

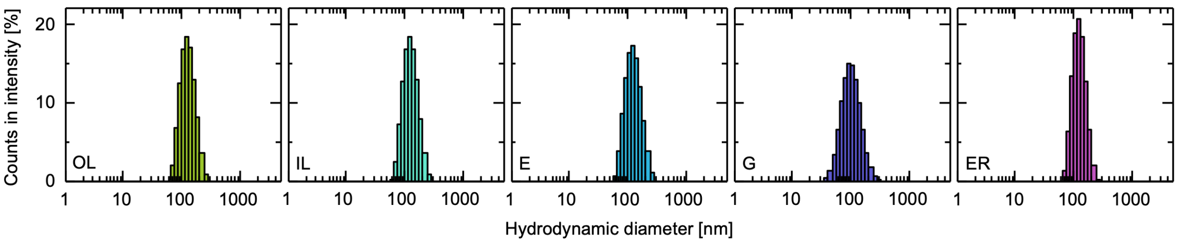

Figure S1. Hydrodynamic diameters of the model LUVs measured by dynamic light scattering. OL: outer leaflet of the plasma membrane (130.7 nm ± 2.3 nm), IL: inner leaflet of the plasma membrane (129.6 nm ± 3.3 nm), E: late endosomes (127.8 nm ± 0.6 nm), G: Golgi apparatus (106.2 nm ± 6.9 nm), ER: endoplasmic reticulum (127.0 nm ± 2.9 nm). 3 independent LUVs preparations were averaged.

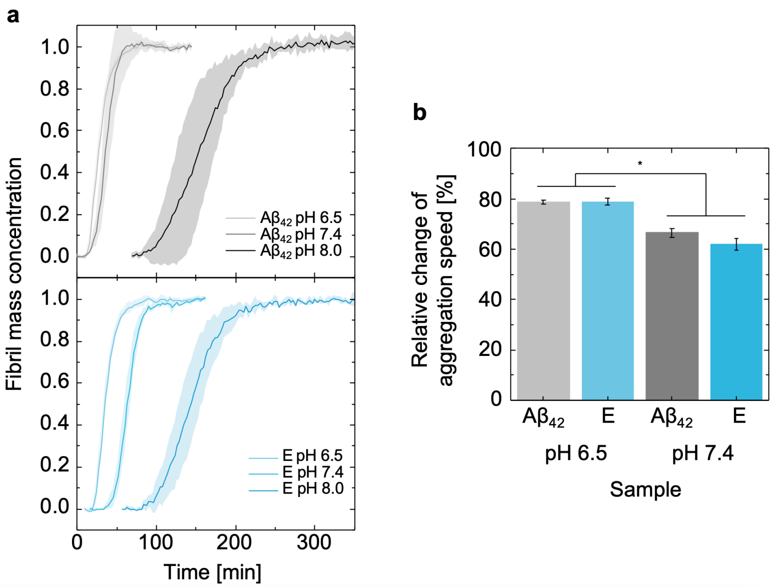

Figure S2. Aggregation kinetics of Aβ_{42} in presence of the endosomal model membranes at different pH values. (a) Set of averaged traces of the Aβ_{42} aggregation kinetics alone (top panel, grey traces) and in presence of the model lipid membranes (bottom panel, blue traces) obtained by a ThT fluorescence assay at pH 6.5, 7.4 and 8.0 (n = 5). **(b)** The half time ($t_{1/2}$) was calculated for each pH value (100 µM lipid concentration) and compared to that of Aβ_{42} in solution (2 µM) at pH 8.0. While the presence of the endosomal model membranes does not affect the Aβ_{42} $t_{1/2}$, decreasing the pH accelerates the aggregation kinetics (acceleration at pH 6.5 statistically significantly greater than at pH 7.4; for the Aβ_{42} pair: $p = 0.0001$, for the E pair: $p = 0.0001$).
